# QTc interval prolongation, COVID-19 and chloroquine

**DOI:** 10.1007/s12471-020-01506-x

**Published:** 2020-10-08

**Authors:** V. Wiwanitkit

**Affiliations:** 1 Dr. D.Y. Patil University, Pune, India; 2 Hainan Medical University, Haikou, China

Dear editor,

I would like to share my ideas on the article titled ‘The risk of QTc-interval prolongation in COVID-19 patients treated with chloroquine’, which was recently published in the *Netherlands Heart Journal* [1]. Herein, Sinkeler et al. reported that ‘Chloroquine treatment in COVID-19 patients gradually increased the QTc interval’ [1]. Indeed, prolongation of the QTc interval is a possible cardiac electrophysiological change in anyone who receives chloroquine [2].

Although I agree with the authors that this requires monitoring, a point to be discussed is the chance that there is a clinically significant QTc interval prolongation. I would like to draw attention to the data from Indochina, where malaria is common and chloroquine is widely used. There are no reports on problems due to QTc interval prolongation among local people who use antimalarial prophylaxis, nor on cardiophysiological disturbances in COVID-19 patients receiving chloroquine therapy. Indeed, COVID-19 might cause arrhythmia *without* chloroquine therapy [3]. Moreover, in the present report by Sinkeler et al. [1], there are no comparative data on QTc interval prolongation in COVID-19 patients who receive another treatment or data from other patients.
